# Epigenetic lifestyle of Epstein-Barr virus

**DOI:** 10.1007/s00281-020-00792-2

**Published:** 2020-03-30

**Authors:** Alexander Buschle, Wolfgang Hammerschmidt

**Affiliations:** Research Unit Gene Vectors, Helmholtz Zentrum München, German Research Center for Environmental Health and German Centre for Infection Research (DZIF), Partner site Munich, Marchioninistr. 25, D-81377 Munich, Germany

**Keywords:** Herpesvirus, Chromatin, Infection, Latency, Reactivation

## Abstract

Epstein-Barr virus (EBV) is a model of herpesvirus latency and epigenetic changes. The virus preferentially infects human B-lymphocytes (and also other cell types) but does not turn them straight into virus factories. Instead, it establishes a strictly latent infection in them and concomitantly induces the activation and proliferation of infected B cells. How the virus establishes latency in its target cells is only partially understood, but its latent state has been studied intensively by many. During latency, several copies of the viral genome are maintained as minichromosomes in the nucleus. In latently infected cells, most viral genes are epigenetically repressed by cellular chromatin constituents and DNA methylation, but certain EBV genes are spared and remain expressed to support the latent state of the virus in its host cell. Latency is not a dead end, but the virus can escape from this state and reactivate. Reactivation is a coordinated process that requires the removal of repressive chromatin components and a gain in accessibility for viral and cellular factors and machines to support the entire transcriptional program of EBV’s ensuing lytic phase. We have a detailed picture of the initiating events of EBV’s lytic phase, which are orchestrated by a single viral protein – BZLF1. Its induced expression can lead to the expression of all lytic viral proteins, but initially it fosters the non-licensed amplification of viral DNA that is incorporated into preformed capsids. In the virions, the viral DNA is free of histones and lacks methylated cytosine residues which are lost during lytic DNA amplification. This review provides an overview of EBV’s dynamic epigenetic changes, which are an integral part of its ingenious lifestyle in human host cells.

## Introduction

Epstein-Barr virus (EBV) is a human herpesvirus with a DNA genome of about 165 kbps [11]. Many known strategies of EBV mimic cellular processes and principles as the virus has coevolved with its human host. EBV has copied central mechanisms of the cell, e.g., receptor signaling, DNA replication, and gene transcription, for its own success. Thus, the viral model is a window to the cell, rich in biology and an excellent source to study epigenetic principles of key importance in metazoan cells.

EBV is also a human tumor virus [5]. It is associated with and contributes to several human tumor entities. Among them are different types of B cell lymphoma (Hodgkin’s disease, Burkitt’s lymphoma, non-Hodgkin’s lymphomas), cancers (nasopharyngeal cancer, gastric cancers), and other malignancies. More than 90% of the world population is infected with EBV for a lifetime, whereas tumor incidence is relatively low, i.e., in the order of 200,000 annually [20, 97]. Certain cofactors contribute to tumor formation, but they are mostly unknown. In all cases, EBV establishes a latent infection in the tumor cells, which express different sets of viral genes, termed viral latency programs.

In vitro, EBV preferentially infects nonproliferating, quiescent human B-lymphocytes, activates them, induces their indefinite proliferation in vitro, and establishes a strictly latent infection in them. In viral particles, the large EBV DNA is epigenetically naïve, i.e., lacks associated histones and is free of methylated CpG dinucleotides. Upon infection, the viral DNA is delivered to EBV’s target cells and maintained as extrachromosomal plasmid copies in the nucleus. Here, the viral DNA acquires nucleosomes including histones with mostly repressive marks and finally a very high degree of CpG methylation. When and how viral DNA acquires cellular chromatin and its individual components and which cellular factors drive this process are largely unknown.

**Fig. 1:**
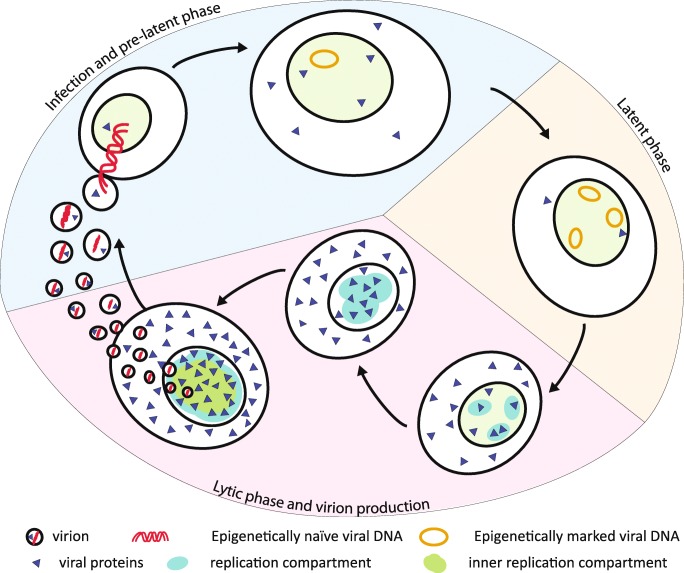
The tripartite life cycle of EBV. Infection and pre-latent phase (light blue segment). Upon infection, a virion releases it epigenetically naïve linear DNA (red) into its host cell. The viral DNA circularizes and makes its way into the nucleus (light green). As the incoming DNA is not epigenetically repressed, many viral proteins (blue triangles) are expressed at low levels initially. During this phase, the host cell grows in size and starts to proliferate (not shown). After a couple of days, histones and nucleosomes are positioned, and mostly repressive epigenetic marks are established on the viral, plasmid-like minichromosome. Latent phase (light yellow segment). EBV expresses only few latent EBV proteins to maintain and preserve its epigenetically repressed DNA in latently infected cells, and EBV DNA acquires a very high level of CpG methylation (not shown). Additionally, this strategy avoids the detection of viral antigens by the immune system. In the nucleus, several copies of viral repressed EBV minichromosomes accumulate via an unknown mechanism. Latent phase and virion production (light red segment). Upon reactivation of the virus’ lytic phase, EBV starts to express lytic viral proteins, repressed viral chromatin opens up, and replication compartments (turquoise ovals) start to form. The full cascade of viral proteins is synthesized eventually, which counteracts the immune response of the host organism, supports lytic amplification of viral DNA, and provides structural proteins such as capsid components, tegument proteins, and glycoproteins. Replication compartments form, grow in dimension, and fuse during the massive replication of viral DNA. The newly synthesized viral DNA is packaged into viral capsid structures within the inner replication compartment (green area within the replication compartment). The newly replicated viral DNA is free of histones and lacks methylated CpG dinucleotides such that assembled virions contain epigenetically naïve viral DNA ready to infect new target cells.

In the so-called pre-latent phase of viral infection [53] during the first 8 days (Fig. 1) when EBV reprograms the resting B-lymphocytes into activated and proliferating B blasts [74], viral transcription is pervasive, which leads to the expression of many viral genes. In contrast, in established latently infected cells, a clearly defined minimal set of very few viral genes is active. It is very likely that the deposition of cellular chromatin onto viral DNA is an essential step in controlling the program of viral transcription of the pre-latent phase ensuring the survival and continuous proliferation of EBV-infected cells long term. As a result, so-called lymphoblastoid cell lines (LCLs) emerge in which EBV establishes a stable latent infection.

Latent infection is a paradigm of all herpesviruses, but EBV excels at latency, its preferred lifestyle. In healthy EBV-positive individuals, B-lymphocytes, in particular a minute fraction of long-lived memory B cells (one in 10^4^ to 10^6^ cells), form the viral reservoir of latent infection. Viral latency has been studied in memory B cells ex vivo, tumor cells obtained from biopsies, and established tumor cell lines as well as in LCLs. LCLs and established tumor cell lines are a rich source to study all aspects of viral latency and have contributed much to understand the molecular mechanisms of viral latency. Certain cell lines such as the Burkitt’s lymphoma cell lines, Akata, Raji, and P3HR1 [48, 85, 103], have also been instrumental to study EBV’s escape from latency ([3, 31, 69, 86, 91]; for a selection of original works) and to identify the viral switch gene BZLF1 [21, 102] that can turn latently EBV-infected cells into virus factories that release viral progeny.

The key to EBV’s success in infecting and persisting in its host lies in its ingenious tripartite epigenetic life cycle (Fig. 1) [45, 117]. We have learned that the virus cannot induce virus de novo synthesis upon cellular infection but enters its so-called pre-latent phase. The virus reprograms the infected B cells, activates them to grow in size, and drives them into several rounds of intense proliferation until proliferation decelerates to a doubling time of about 30 h. In these cells, which are now in the latent phase, no virus is synthesized, but a small subset of viral genes is expressed, and several copies of the viral genome are maintained as extrachromosomal plasmids. Upon induction of EBV’s third and lytic phase, the full set of about 80 viral genes is expressed. The viral genomic DNA replicates autonomously, and viral progeny is synthesized. Infectious viral particles are released and spread horizontally to other permissive cells or are transmitted to other individuals.

Epigenetic principles govern the different expression patterns linked to the three phases of EBV’s life cycle (Fig. 1). The virus has evolved to take advantage of the host cell’s epigenetic machinery to first establish a stable latent infection and then to use a smart principle to escape from it.

## EBV’s virion DNA is epigenetically naïve

In viral particles, the large EBV DNA is epigenetically naïve, i.e., it lacks associated histones [54] and is free of methylated CpG dinucleotides ([60], Supplementary Fig. S3 in ref. [57]). This is a surprising finding but fully in line with other herpes viruses [39]. We will see later how EBV achieves this state, which is reminiscent of epigenetic events in sperm DNA during fertilization.

Sperm DNA is mostly free of nucleosomes, and compared with somatic cell DNA, the levels of histone retention are 1 and 10% in mouse and man, respectively [12]. Shortly after fertilization and prior to DNA replication or transcription in the fertilized egg, the histone variant H3.3 is incorporated by the histone chaperone HIRA. This step precedes the genome-wide demethylation that takes place in the male pronucleus. Prior to DNA replication, H3 and H4 histones in paternal DNA, which are newly acquired from the oocyte, are acetylated, and H3 histones gain mono-methyl marks at K4, K9, and K27. Only after the first round of DNA replication, paternal DNA acquires tri-methylation of some of them, notably H3K4me3, and H3K27me3 [92].

Why could this information be important for the first steps in EBV infection? It seems plausible that certain aspects are recapitulated by EBV upon infection of primary B-lymphocytes. For example, the incoming EBV DNA rapidly acquires nucleosomes in the infected cell prior to its first round of DNA replication and cell division. The molecular mechanisms are unknown, but the viral model offers an attractive, perhaps even unique, opportunity to investigate de novo chromatin assembly and regulation in a tractable system and in mammalian cells. Parallels in viral DNA in the initial phase of infection and in sperm DNA in zygotes with regard to de novo establishment of chromatin suggest that both fundamental processes might be mechanistically and biologically similar.

## Acquisition of cellular chromatin components upon infection

Little is known how EBV DNA acquires chromatin constituents upon de novo infection. Presumably, the earliest action is the recruitment of histones supported by histone chaperones such as HIRA, DAXX, CAF1, ASF1, DEK, and several others, which can manage transport, location and deposition, mobilization, and replacement of nucleosomes. To our knowledge, close to nothing is known about the timing and dynamics of this step during EBV infection. We only know from preliminary experiments that the landscape of nucleosomes on EBV DNA is completed in B-lymphocytes within 48 h post infection (Mrozek-Gorska et al., unpublished). This observation suggests that nucleosome assembly is replication independent and occurs in activated cells during G1 phase and long before the onset of EBV-induced cellular DNA replication [74]. Based on this assumption, it is likely that replication-independent histone chaperones are predominantly involved in this early step of chromatinization.

It is enigmatic what the initial steps of histone deposition on EBV DNA are and where the histones come from in the very early phase of infection. Canonical histones are produced only during the DNA synthesis (S) phase of the cell cycle, suggesting that the available histones might be noncanonical histone variants such as H3.3 or histones that belong to the small pool of free but chaperone-complexed, stored histones. Alternatively, histones are recycled in this early phase of viral infection, because the expression of two viral key activators, EBNA2 and EBNA-LP [58, 113], within the first 48 h of infection [74] induces global cellular gene expression (ibid.) that likely evicts nucleosomes from cellular chromatin. They might be available to be positioned onto the incoming naïve EBV DNA.

Clearly, histone chaperones meet the demand of de novo histone deposition to support diverse nuclear processes including the masking and gap-filling of EBV’s naked genomic DNA. Among the many potential histone chaperones [47], HIRA, histone regulation A, a chaperone for the histone variant H3.3, seems to be an interesting candidate as it is involved in replication-independent histone deposition (together with DAXX and its co-chaperone ATRX). The HIRA complex has also been reported to act as gap filler strongly suggesting that it can interact with naked DNA [90]. In addition, HIRA is required for male pronucleus formation, which is inhibited due to a lack of nucleosome assembly in the sperm genome in the absence of HIRA [68]. HIRA is the only H3.3 chaperone that is incorporated broadly into decondensed sperm DNA at fertilization [68], which seems to be in line with the acquisition of a nucleosomal structure in EBV DNA.

Solid data are available for DAXX, also a chaperone for the histone variant H3.3, and its role in EBV infection. DAXX is bound by BNRF1, a tegument constituent and a member of a class of related proteins found in gamma herpesviruses [109] to promote selective viral gene expression [108, 110]. The binding of BNRF1 displaces the interaction of DAXX with the SWI/SNF-like chromatin remodeler ATRX [110]. ATRX directs DAXX to heterochromatin loci as ATRX specifically recognizes H3K9me3, a suppressive histone mark (see references in [47]). Surprisingly, virion-contained BNRF1 protein reduces the deposition of histone H3.3 onto viral DNA by DAXX and enhances viral gene expression early during EBV infection [50, 108]. This finding is interpreted to mean that BNRF1 prevents the deposition of histones and the formation of nucleosomes exclusively at promoters of EBV’s latent genes that support B cell activation and transformation long term. It thus appears as if DAXX acts downstream after the initial formation of nucleosomes on EBV DNA to prevent epigenetic repression of critical latent genes of EBV.

Viral transcription appears to be pervasive early after infection in the pre-latent phase, because transcripts of lytic genes are found expressed although mostly at low levels. Among them is BZLF1 [57, 114] but also many others as revealed in a global transcriptomic approach ([74], see also the web tool http://ebv-b.helmholtz-muenchen.de/). It thus seems that the epigenetically naïve EBV DNA, which lacks histones and nucleosomes and is free of methylated CpG dinucleotides, serves as a permissive template for the cellular transcription machinery. This promiscuous state of transcription comes to an end presumably when EBV DNA acquires cellular nucleosomes, which restricts DNA accessibility, similar to other members of the herpesvirus family [2].

## Stable viral latency

We know much about the epigenetic state of latent EBV infection in B cells. As a rule, several copies of EBV’s genomic DNA are maintained as stable, extrachromosomal plasmids in latently infected cells ([26] for a recent review). The genome copies adopt a genuine epigenetic signature that is accomplished by the host cell. Viral latency is ensured by a strong epigenetic repression of lytic genes, while active latent viral genes are spared depending on the cell type. Repression is implemented and maintained by CpG methylation of viral DNA, by high-density packaging of nucleosomes, and by Polycomb group proteins that establish and maintain the key repressive modification H3K27me3 on EBV chromatin [116]. The repressive H3K9me3 histone mark is also detectable at low levels, but in contrast to H3K27me3, H3K9me3 is not removed upon viral reactivation suggesting that this mark is not central to maintaining viral repressive chromatin (see the chapter “Lytic reactivation” below). The epigenetic signature of viral chromatin has been characterized and documented [7, 89, 106, 116].

CpG methylation of viral DNA is another layer of EBV’s epigenetic regulation during latency. Surprisingly, the acquisition of CpG methylation is a very slow process in newly infected B-lymphocytes. Genomic EBV DNA in virions is virtually free of 5′-methylcytidine nucleotides, and CpG-methylated residues are not detectable immediately after infection (Fig. S3 in [57]). The viral DNA slowly acquires cytidine methyl groups, a process that takes weeks to completion [57, 60]. The slow kinetics suggest that CpG methylation of EBV DNA is not essential to downregulate lytic viral gene expression during the pre-latent phase of infection but might contribute to stabilize lytic gene repression long term. Clearly, EBV DNA reaches a very high level of CpG methylation, eventually, with a methylation rate of close to 100% in vitro in LCLs (Supplementary Fig. S1B and Table S1 in [116]) and in latently infected memory B cells in vivo (Fig. 1E in [116]). Most likely, de novo methyltransferases such as DNMT3a and b and the key maintenance methyltransferase DNMT1 introduce and maintain CpG methylation in genomic EBV DNA, respectively. How these enzymes are regulated during infection and how they get access to viral DNA are elusive.

Recent reports have studied the genomic organization of latent EBV genomes. The chromatin in different EBV cell lines and latency types differs [101], and also the three dimensional architecture of the EBV genome varies in cells in which the virus establishes different latency programs [7, 105]. The three-dimensional adjustment of the chromatin fiber is an important mechanism of gene regulation. Looping and bending of DNA and connecting or insulating *cis*-acting elements are also main principles of enhancer regulation. The ring-shaped cohesin complex is involved in the regulation of chromatin architecture in mammalian cells (summarized in [112]). The DNA-binding protein CTCF, the mediator complex, and the protein complex cohesin are central factors involved in these processes. Mediator and cohesin can connect enhancers to certain promoters over a long distance leading to their transcriptional activation [55]. Vice versa, CTCF and cohesion can insulate a promoter from an enhancer element promoting gene silencing [80, 115]. Cohesin sites have recently been shown to be involved in the establishment of transcriptional memory by guiding transcription factors to their binding sites [118]. Moreover, cohesin has been also reported to act as a key structural element to regulate chromosome-wide gene expression [23].

Thus, it is not surprising that latent EBV chromatin is also organized via CTCF and cohesin subunits. There are close to twenty sites where CTCF and cohesin bind and were mapped in EBV’s latent chromatin ([6, 7] and own unpublished findings), and certain CTCF sites have been implicated to act as insulators. They have been reported to prevent the spreading of repressive marks and progressive CpG methylation of viral DNA and keep latent viral promoters in an active state [106]. EBV’s DNA is organized in loops [105] suggesting that higher-order chromosome conformations might be important to regulate levels of latent viral genes [67].

In the nucleus of latently infected cells, several copies of EBV’s genomic DNA are maintained as stable, extrachromosomal plasmids, which replicate via *oriP*, the viral plasmid origin of DNA replication ([19, 26] for two recent reviews). *oriP* is an important regulatory element that also acts as a viral enhancer to coordinate latent gene expression [38, 79]. Its function is under the control of EBNA1, a latent viral factor that binds *oriP* and likely ensures prominent nucleosome-free or poor regions within two parts of *oriP*, the family or repeats and the dyad symmetry element [7, 72]. As a consequence, neighboring nucleosomes contain histones with activating marks such as H3K4me3, H3K27ac, and H3K4me1 (unpublished data) suggesting that *oriP* is an island of euchromatin to support transcription of latent genes such as the latent membrane proteins and several EBNAs in an otherwise heterochromatic and epigenetically repressed EBV genome. Interestingly, *oriP* also mediates a molecular link to host cell chromosomes suggesting that it acts as an anchor to tether EBV’s genomic copies to nuclear chromatin [73]. Again, EBNA1 is the critical viral factor in *trans*. In latently EBV-infected cells, it mediates tethering of viral DNA to cellular perichromatin [28] and ensures the maintenance and partitioning of viral genome copies in resting and proliferating cells, respectively [19].

## Lytic reactivation

In the latent phase, all lytic genes are strictly repressed, but the ectopic expression of a single viral gene, BZLF1 (also called EB1, ZEBRA, Z, or Zta), can induce the full lytic cycle in certain cells in vitro [21, 102]. In latently infected B cells in vivo, BZLF1 is expressed when memory B cells encounter their cognate antigens and terminally differentiate into plasma cells, a process that supports virus de novo synthesis [62, 107]. Also, in vitro activation of the B cell receptor is clearly linked to viral reactivation in certain established cell lines latently infected with EBV ([4, 98] for earlier reviews [111, 121] for recently published work). Viral micro RNAs counter-regulate downstream signals of the B cell receptor and interfere with lytic phase induction suggesting that B cell receptor triggering is an important route to escape from latency [17]. The regulation of BZLF1 transcription downstream of the activating signaling cascade is complex and involves numerous cellular transcriptional activators and repressors, chromatin constituents, and histone modifications to activate the repressed BZLF1 gene. Regulated expression of BZLF1 has been the subject of several recent reviews [37, 59, 76] and recent original work [69]. Not surprisingly, repression of BZLF1’s promoter appears similarly complex and multifaceted. The structure and epigenetic landscape of the BZLF1 promoter differ from the majority of EBV’s lytic promoters of the so-called early class of viral genes as it contains only very few CpG dinucleotides, which are even spared from cytosine methylation in vitro and partially also in vivo [116] suggesting that BZLF1’s repression is probably not controlled by CpG methylation.

Upon induced reactivation or ectopic BZLF1 expression, the second essential early lytic gene, BRLF1, is expressed. Both genes are indispensable for the lytic phase [33] and encode transactivators that activate viral and certain cellular promoters, leading to an ordered cascade of viral gene expression: activation of early gene expression followed by the lytic cascade of viral genome replication and late gene expression.

## BZLF1 is EBV’s lytic switch

Much is known about BZLF1 and its molecular functions. Briefly, BZLF1 is a member of the AP-1 family and a bZIP protein, binds viral and cellular DNA sequence specifically as a homodimer and even in nucleosome-dense compacted chromatin, activates transcription, acts as a viral pioneer factor, recruits chromatin remodelers that open repressed chromatin locally where BZLF1 binds, has a peculiar preference to bind to CpG methylated DNA sequence motifs, and is indispensable for lytic viral DNA replication as it recruits viral replication factors to EBV’s lytic origin of DNA replication, *oriLyt*. Many of BZLF1’s molecular functions have been detailed, recently.

The atomic structure of BZLF1 bound to DNA has been resolved [49, 83]. It shows the binding of BZLF1 to two different classes of related sequence motifs [8, 9, 32, 119] with two rather different dissociation constants [8]. Paradoxically, BZLF1 preferentially binds to methylated-binding motifs, commonly called meZREs, such as 5’-TGAGmeCGA-3′, which are prevalent in highly CpG methylated promoters of viral early genes in EBV DNA. These sequence elements contain two 5′-methylcytosines (meC) in the top and bottom strands. BZLF1 was the first example of a sequence-specific transcription factor that preferentially recognizes and selectively binds methylated cytosine residues within a specific sequence. This exceptional feature is essential for this herpesvirus to escape from its latent phase of infection [56, 57].

Upon induction in latently infected cells, BZLF1 induces downstream viral promoters of several early genes some of which encode essential components of the autonomous DNA replication machinery of EBV’s lytic phase [34, 35]. The promoters typically encompass CpG dinucleotides in high density and clusters of meZRE sites, which have to be methylated to be bound by BZLF1 to induce these early genes [8, 56, 57, 87, 116, 119]. At low levels, BZLF1 stably binds to meZREs in viral and cellular chromatin in contrast to non-meZREs sites that BZLF1 only binds efficiently at high BZLF1 levels [8, 14]. For example, the promoters of the early lytic BBLF4 and BBLF2/3 genes, which are both essential for lytic viral DNA amplification [34, 35], contain numerous meZREs, which are preferentially bound when methylated. Only then can BZLF1 activate these promoters efficiently [8].

BZLF1 also needs to bind certain low affinity sites in the viral lytic origin of DNA replication, *oriLyt*, where BZLF1 acts as an essential replication factor [94, 95]. This aspect suggests that BZLF1 has to reach a considerably high threshold level to support EBV’s lytic phase fully (see below). Moreover, expression of many late viral genes depends on amplified genomic DNA templates that result from lytic viral DNA replication only ([15, 29, 41]; reviewed in [42]). It thus seems that critical BZLF1 levels directly and indirectly control the ordered and timely lytic expression of all viral genes. Autoregulation of BZLF1 expression via a positive feedback loop [98] and non-meZRE sites and meZRE sites (in the promoters of BZLF1 and BRLF1, respectively) might be a prerequisite to achieve sufficiently high BZLF1 levels to complete EBV’s lytic phase [10, 36, 98].

Recently, characterized molecular functions of BZLF1 suggest that this viral protein is a bona fide pioneer transcription factor [120] that has direct access to epigenetically repressed (viral) chromatin inducing its transcriptional competence. Upon sequence-specific binding even in densely packed nucleosomal EBV DNA [93], BZLF1 unfolds its potential as a pioneer factor preferentially on meZRE sites [116]. BZLF1 recruits chromatin remodelers such as INO80 and probably other abundant remodelers that evict local nucleosomes providing access to promoters and *cis*-regulatory elements in viral chromatin [93]. As a consequence, nucleosomes at BZLF1-binding sites are lost, and repressive chromatin marks such as H3K27me3 on flanking histones are erased, while H3K9me3 marks are not affected [75, 89, 116]. Concomitantly, previously bound Polycomb group proteins (PCG) and the writer EZH2 are no longer associated with viral DNA [116]. Induction of EBV’s lytic phase eliminates the repressive H3K27me3 mark on lytic promoters relieving their tight repression. The resulting open chromatin allows loading of the RNA polymerase II (RNAP II) multiprotein complex with its transcriptional machinery. Active histone marks such as H3K4me3 are set especially at early lytic promoters [116] and trigger EBV’s escape from latency. Additionally, the interaction of BZLF1 with the histone acetyltransferase CBP increases the transactivation of early EBV promoters [1, 27]. The subsequent acetylation of histones in the promoters of BRLF1 and BZLF1 correlates with their expression [16, 52, 75] but is not necessarily sufficient to induce them [22, 40]. Unexpectedly, CpG methylation of viral DNA is maintained throughout this early viral phase of transcriptional reactivation and is no hindrance to active transcription of extensively CpG methylated viral genes as thought previously [116].

In contrast to the consequences of BZLF1 binding to viral DNA, alteration of the chromatin architecture of EBV’s genomic DNA and impaired functions of the chromatin organizer CTCF, which organizes the viral genome during latency and regulates the viral programs of latent transcription ([82] for a recent review), do not result in lytic reactivation [69].

Upon lytic reactivation, the EBV minichromosomes move within the nucleus from their pericentric positions [28] with gene-poor, AT-rich, and repressive heterochromatin to active euchromatin [73]. EBV’s genomic DNA remains attached to the nuclear matrix as during the latent phase of infection, but the point of attachment changes upon lytic reactivation from the plasmid origin of DNA replication *oriP* [51] to *oriLyt* sequences [71]. It seems as if EBV positions its genomes in a higher-order nuclear context reflecting its requirements for efficient expression of its genes.

EBV not only governs the cellular machinery to reverse epigenetic repression of the viral genome but also induces a genome-wide reorganization and alteration of the cellular epigenome and transcriptome. As in viral DNA, BZLF1 binds the same two major sequence motifs in cellular chromatin [14, 88]. More than 190,000 (± 40,000) BZLF1-binding sites are identified [14] throughout cellular DNA [81, 104]. While the induced expression of BZLF1 in EBV-negative cells causes only minor alterations of cellular gene expression, the expression of BZLF1 in latently infected B cells results in a massive reduction of the cellular transcriptome and profoundly alters the cellular epigenome within 6 to 15 h post induction [14]. Regions of previously open and accessible chromatin close genome-wide. Concomitantly with the reduction of open chromatin, middle- and long-distance chromatin interactions between promoters and their interacting regulatory regions are strongly reduced [14]. EBV’s objective of these alterations is not understood in the moment, but already Adamson et al. speculated in 1999 that the interaction of BZLF1 with CBP might interfere with the availability of histone acetyltransferase CBP at transcriptionally active cellular loci [1]. Within 1 day of lytic phase induction, cellular chromatin was found to become highly condensed, the nuclear lamina was redistributed [63], and cellular histones appear to move to the periphery of the cell nucleus [18].

The purpose of restructuring cellular chromatin is not obvious but might have its origin in creating space for nuclear sites where lytic amplification of viral DNA takes place. The nuclear structures that are termed amplification or replication factories (or compartments) [63] form in lytically induced cells infected with EBV [104] or other viruses (reviewed in [96]). Viral proteins known to associate with the lytic replication of viral DNA accumulate in EBV’s replication compartments, which are microscopically visible structures (Fig. 1 in [24]) where viral DNA replicates and amplifies massively [78].

## Loss of chromatin constituents and DNA methylation upon lytic amplification of viral DNA

The replication compartments appear within the nucleus seeded by single viral genomes [78] and increase in size over time, fuse, and finally occupy large parts of the nucleus (Fig. 1 in [24]). When EBV’s DNA is amplified in the replication compartments and becomes microscopically detectable, it does not co-localize with histone chaperones nor with canonical or noncanonical histones such as H2B, H3.1, or H3.3 [18] indicating that cellular machines that deposits histones on newly synthesized cellular DNA are excluded from viral replication compartments. Lack of nucleosomal structures alters the superhelicity of DNA, and in fact, viral DNA was found to be less supercoiled in this phase compared to DNA extracted from latently infected cells [84] supporting this view.

Viral replication compartments are operationally subdivided into outer [“ongoing replication foci”] and inner [“BMRF1-cores”] domains [100], in which early and late lytic genes are transcribed, respectively [99], while transcription of late lytic genes is only conducted simultaneously with the replication of and from newly synthesized viral genomes free of nucleosomes ([29, 30, 64]; reviewed in [15]). While these findings might give the impression that unmethylated viral DNA is required for late lytic gene expression, demethylation of viral DNA alone is not sufficient [64]. The molecular link between herpesvirus lytic DNA replication and late gene transcription is interesting and has been reviewed very recently [15, 42].

Within the outer domain of replication compartments, ongoing de novo viral DNA synthesis is accompanied by cellular homologous recombination repair proteins, while cellular mismatch repair proteins were found in the inner domain, where the newly synthesized DNA seems to accumulate [25, 61, 100].

Also excluded from lytically replicating herpesviral DNA in replication compartments is proliferating cell nuclear antigen (PCNA) [77]. PCNA is essential for eukaryotic licensed DNA replication (and DNA repair) and acts as a trimeric DNA sliding clamp and processivity factor for DNA polymerase δ in eukaryotic cells and as a scaffold to recruit proteins involved in DNA replication, DNA repair, chromatin remodeling, and epigenetics. Among the recruited proteins is the cellular DNA methyltransferase 1, DNMT1, that together with UHFR1 couple semiconservative cellular DNA replication and DNA methylation such that newly replicated daughter strands inherit the pattern of CpG methylation of parental DNA. In cells that support EBV’s lytic phase, PCNA localizes to the amplification factories, but PCNA is not detected at the sites of viral DNA synthesis [18]. This is no problem for the virus because it encodes its own autonomous viral factors that mediate lytic herpesviral DNA replication independent of the host cell [34, 35]. The function of PCNA in lytic DNA replication of EBV is replaced by the structural homolog BMRF1 [46]. In the absence of PCNA, DNMT1 is not recruited to the replication forks of herpesviral DNA, and methylation marks at cytosine residues are lost on both newly synthesized DNA strands, removing this epigenetic modification from EBV’s genomic DNA. To our knowledge, this scenario has not been formally proven yet, but it is highly plausible ([18, 57] and references therein). As a consequence, the isolated packaging of the amplified viral DNA into capsids ensures that EBV’s genomic DNA within virions is free of methylated CpG dinucleotides ([60], Supplementary Fig. S3 in ref. [57]).

In summary, EBV prevents the loading of cellular histones on its newly synthesized DNA [54] and uncouples viral DNA replication from the activity of DNMT1 that maintains the methylation pattern in the newly synthesized strand in cellular DNA [56]. Both strategies ensure that EBV’s DNA in virions is epigenetically naïve when the virus launches its next epigenetic life cycle in a newly infected cell. Surprisingly, BZLF1 will be detectably expressed in the newly infected cell within hours [44, 57, 114], presumably because the epigenetically naked viral DNA does not prevent an initial, promiscuous, and genome-wide transcription [53]. Nevertheless, BZLF1’s transient expression fails to initiate EBV’s lytic phase in these newly infected cells. The lack of CpG methylation of EBV’s virion DNA seems to be an important reason because unmethylated meZRE sequence motifs will not allow an efficient BZLF1 binding preventing the onset of the cascade of lytic viral genes from the start [117]. The abundance of cellular BZLF1-binding sites might be a second fail-safe mode to mitigate the accidental induction of EBV’s lytic phase in freshly infected cells [14] indicating that latency is definitely EBV’s preferred lifestyle.

## Open questions

### Chromatin acquisition when, how, and by whom

It is unknown what drives the very rapid nucleosome formation on the incoming EBV DNA very early after infection of primary human B-lymphocytes. There are assumed players such as histone chaperones, but this area is rather speculative. It might be rewarding to invest here, because this viral model offers an attractive, perhaps even unique, opportunity to investigate de novo chromatin assembly and regulation in a tractable system and in mammalian cells. The epigenetic mechanisms of developmental programming during fertilization in the zygotic state and in the early embryo are technically difficult to study [13], but the EBV infection model is biochemically and genetically accessible, which seems to be attractive.

### Polycomb recruitment and timing of H3K27me3 histone marks

It remains elusive how EBV DNA acquires nucleosomes and when they turn into repressed chromatin as indicated by characteristic H3K27me3 histone marks (among others). Similar to EBV, its closest relative, Kaposi sarcoma-associated herpesvirus (KSHV), can also establish latent infections in certain cell types. Recently, in a collaborative work, Adam Grundhoff has studied the kinetics of Polycomb repressive complexes (PRC) on KSHV DNA in infected cells and found a correlation between the recruitment of the noncanonical PRC1.1 component KDM2B, CpG islands, and H3K27me3 marks suggesting that high local concentrations of unmethylated CpG clusters attract Polycomb complexes to implement chromatin repression on viral DNA [43]. KSHV differs from EBV as KSVH chromatin adopts a bivalent mode in its latent phase. Therefore, it remains to be investigated how and when EBV’s DNA acquires typical repressive marks indicative of Polycomb functions.

### Epigenetics and EBV-associated malignancies

EBV is associated with or even causes very different types of malignancies in man. Latent viral gene products are likely contributors to oncogenic processes in these tumor cells, but also lytic EBV genes have been blamed to be involved in carcinogenesis. Even in tumors that are predominantly latently infected such as nasopharyngeal carcinoma, for example, fractions of cells enter the lytic phase perhaps playing a contributing role to tumor formation [66, 70]. Immune responses to both latent and lytic viral gene products are manifold, broad, and rather diverse, but the optional induction of the lytic phase in tumors cells likely turns them into better targets for cellular immunity [66]. It is intriguing to think that the manipulation of the epigenetic program in EBV tumors in vivo might provide a therapeutic window as discussed recently [65].
